# Marital satisfaction and adherence to religion

**Published:** 2015

**Authors:** F Jafari, L Neisani Samani, N Fatemi, S Ta’avoni, J Abolghasemi

**Affiliations:** * School of Nursing and Midwifery, Tehran University of Medical Sciences, Tehran, Iran; ** School of Nursing and Midwifery, University College International of Medical Sciences, Tehran, Iran; *** Iran University of Medical Sciences, Tehran, Iran

**Keywords:** marital satisfaction, religion statement of problem

## Abstract

**Background:** One of the most important determinants of health and marital satisfaction, the family and religious adherence can be effective because religion includes guidelines for life and providing a system of beliefs and values make these features can affect family life.

**Approach:** This descriptive research - an analysis performed to assess the level of satisfaction of 47 questionnaires marital satisfaction questionnaire whose validity and reliability were evaluated and a couple of them asked to assess adherence to religion. The study population included 382 couples in Tehran that a cluster of 22 districts of Tehran were the selected. To analyze the data, ANOVA, Chi-square, and Pearson correlation coefficient using the software SPSS (version 22) became all tests were performed at the 5% level.

**Results:** The data showed that the average age is 34 for women and 38 years for men and the majority of couples are in appropriate level in religiosity (40.5 percent). The results showed a main direct relation among religiosity and marital satisfaction of men and women (p ≤ 0.001). The correlation among religiosity and marital satisfaction of women r = 0.271 and this factor in men r = 0.200 was obtained indicating a direct relationship was significant.

**Conclusion:** couples who were both committed to religion, their marital satisfaction score was more than couples without adherence to religion, and thus promoting religious beliefs and commitment can increase their marital satisfaction in couples.

## Introduction

The Holy Quran is the word of the couple’s marital satisfaction and a sense of peace and calm are the result of knows it (I: 21) that will be affected by internal and external factors. The peace and happiness will be achieved when having a good relationship with and adhere to the rules of origin and divine commands (Munafiqun: 9). Marital satisfaction is an important aspect of personal health. Relationship with partner central aspect of a person’s emotional and social life and marital satisfaction can be the inability to establish consensual couples with children and other people outside the family damage [**1**]. 

Negative and irreversible effects on mental health and substance couples leave [**2**]. A variety of factors have been identified as factors influencing marital satisfaction that some of these factors include: age at marriage, duration of marriage, children, the proportion of academic, financial, sexual, relatives and acquaintances, religious beliefs, personality traits; ability wife; communication skills; leisure and commitment [**3**]. In the religious orders, having common is the important factor. Meanwhile, religious beliefs and values are the most important factors in the stability of marital life [**4**].

In fact, religion is a set of beliefs, including moral values, traditions, participate in the religious community for the conviction to a higher power. Religious beliefs are an effective way to deal with disasters, disease symptoms (Army, 2010) is the result of a lack of religious commitment can be made without a commitment to behaviors that lead to a decrease in marital satisfaction of spouses [**5**]. Several studies have been done to the satisfaction of Islam. Based on this research, religious values are predictor of marital success and continuity of marriage and thereby targeted to people’s lives. Nazari believes that religious values about marriage are important for mental health [**6**]. Marital and religious values can be shared semantic basis for meaning to life [**7**]. Hanler and Knchoz also believe that the religious attitude can be effective satisfaction because religion provides guidelines for life and system of values [**8**]. Kim and others in their research showed that the practice of religious beliefs has a positive relationship with emotions and positive emotions, like a good mood, happiness, kindness, confidence, respect and peace [**9**].

Corporate, after his studies to the conclusion that religious disputes among family members cause problems for the marriage to be a problem to reduce conflicts and marital satisfaction and marital discord, and divorce takes [**10**]. The results of Rohani and Manavipour’s research (2008) which examine the link among the training of religious opinions and marital satisfaction and happiness, they have found that religiosity has significant positive correlation with marital happiness and satisfaction. Mullins and colleagues in a study to examine the relationship between religious belief to practice their marital satisfaction and happiness. Results showed that religion significantly correlated with the amount of love and happiness wives [**11**]. Johnson and colleagues concluded in their study that religion is a decrease in marital conflicts, and improved mental health [**12**].

Ghafuri’s study (2009) showed that adherence to religion is an important factor in marital satisfaction and differences in religion lead to disputes [**13**] showed that religious beliefs has a direct impact on marital satisfaction, but [**14**] argues that the relationship between adherence to religious beliefs and marital adjustment was not significant. In a study [**15**] conducted on 100 couples in Tehran, they concluded that, there was a clear positive link among the practice of religious beliefs and marital satisfaction. In this study, the two researchers have reported that their level of education on religious orientation and religious beliefs have a significant impact, while gender had no significant effect on religious orientation and religious beliefs. This means that people with high education level had a positive approach towards religion and religious beliefs was their [**16**].

Marital satisfaction shelter, a healthy environment for physical and mental health of spouses and family members provide a foundation that can future generations, the development of society, culture and transmitting values where possible [**17**]. Researchers believe that since religion and family are closely related to each other, strengthen each of which can strengthen marriage and the marital satisfaction [**18**]. In this regard, the researcher aimed to study the influence of religious commitment on their marital satisfaction.

## Methods

This is a research on 382 couples of childbearing age living in 22 districts of Tehran, in 2015. Inclusion criteria included all healthy and married couples of reproductive age living in Tehran who were eligible for the following: Lack of affection couples to infertility, disease, life-threatening, chronic and incurable physical and psychological According to the same study, no incidents of tension in the 3 months prior to the sampling, the women not in pregnancy and breast-feeding exclusively, women with 15 to 49 years old, drug abuser spouses, alcohol and tobacco, passing at least one year of marriage, both families and couples. Sample size according to research conducted in the same areas, the average of marital satisfaction was declared in couples between 95 and 153 between 8.89 and 9.5 with a standard deviation and by taking into account 95% confidence level with a 15 percent probability sample, the sample size of 382 was calculated Couples [**19**].

The questionnaire was used to collect data. In the first section, demographic information and asked adherence to religion. In the second part of the tool, Enrich Marital Satisfaction, there were 47 questions. Enrich a measuring scale test of the distorted ideal of marriage, marital, association, personal issues, financial administration, conflict solution, leisure actions, sex, kids, parenting, friends and family, egalitarian roles related to gender, orientation - religious solidarity married couples and changes to be included. Answering the questions was as follow: opposite quietly (1), against (2) neither agree nor disagree (3) agree (4), strongly agree (5). The survey is given to each option from 1 to 5 points and eventually meets after the scores are added together.

The validity and reliability of Enrich have been approved in several studies. Many researchers, such as Mahdavian in Iran (1997), Moradi (2001), Sanaei (2000) have shown that the scale Enrich and Persian translations of validity and reliability are required. Olson and others (1989) recent form validation using alpha 92/ 0 reported. Also in this study, firstly, the questionnaire was approved by 10 professors at the Nursing and Midwifery School and the Medical Sciences University, and secondly with a pre-test was completed for 20 couples of reproductive age, and reliability (Cronbach’s alpha) for marital satisfaction was calculated as 91.9 that is valuable.

Multi-stage cluster sampling was conducted. 22 districts of Tehran, the similarity of cultural, social, economic and level of development were divided into 5 groups. In the first phase of each of the 5 groups in the city, an area as clusters were selected by lottery. The regions included were 6, 5, 7, 10, and 19. In the second stage of each of the 5 clusters, three quarters and in every neighborhood, three streets at random with the help of a satellite map of Tehran, Tehran’s municipality provided that the site was chosen. In the third stage due to the sample size in each street and house number was randomly chosen street, and each of the couples were given questionnaires to complete them [**20**,**21**].

## Results

The results showed that the majority of couples were among 22 and 35 years, percentage of females in this category is more than men. The average age of women is 34 and men is 38 years old. The results showed that the majority of couples were between the ages of 22-35 years. Almost all men age at the time of marriage was three years older than their wives (1.38 percent). One couple had a child and (3.6%) of couples have had 4 children and more, but on average each family had two children. Most of the women and men surveyed (62.8 percent) have a college education and a significant difference was seen between the education of women and men surveyed (58.7 percent) were housewives and (47.6 percent) were male employee.

**Table 1:** Correlation coefficient of marital satisfaction and adherence to religion

Adherence to religion of husbands	Adherence to religion of wives	Marital satisfaction of husbands	Marital satisfaction of wives	
			1	Marital satisfaction of wives
		1	0/**701	Marital satisfaction of husbands
	1	0/**225	0/**271	Adherence to religion of wives
1	0/**661	0/**200	0/**267	Adherence to religion of husbands
2/22	2/28	155/15	156/80	Mean
0/680	0/684	20/891	22/818	SD
**- Correlation coefficient was significant at the error level of 0.01				

To investigate the link among religiosity and marital satisfaction of spouses of women Pearson correlation test was used. The findings indicated that the there is a clear direct link among marital satisfaction of wives and husbands (0.701), religiosity wives and husbands (0.661), women between religiosity and marital satisfaction (0.271) and marital satisfaction of spouses (0.225), as well as men between religiosity and marital satisfaction for women (0.267) and marital satisfaction of spouses (0.200). The analysis of variance was used for the contribution of religious commitment in anticipation for women and their husbands’ marital satisfaction.

**Table 2:** Average satisfaction in terms of adherence to religion in both sexes

Husbands		Wives		Marital satisfaction / Religion Adherence
Mean ± (SD)	Freq.	Mean ± (SD)	Freq.	
96/ 150 ± 384/ 16	54	97/ 155 ± 196/ 21	39	low
72 /155 ± 225/ 19	183	98/ 153 ± 616/ 21	187	med
18/ 162 ± 837/ 21	138	38 /171 ± 653/ 23	144	high
-	7	-	12	No response
157/41 ± 20/20	382	163/47 ± 22/23	382	Total

**Table 2** shows that an increased adherence to religion in both male and female increases sexual satisfaction.

**Table 3:** Marital satisfaction survey subjects in terms of adherence to religion

Sig.	Statistics	Mean of square	Degree of freedom	Total squares	Statistics /Source of variance	
001/ 0	7/484	875/ 2953	2	750/ 5907	Intra-group	Wives
		691/ 394	372	184/ 146825	Intergroup	
000 /0	026/ 15	873/ 7532	2	746/ 15065	Intra-group	Husbands
		331/ 501	367	427/ 183988	Intergroup	

**Table 3** shows the results of analysis of variance, given that a significant predictor of marital satisfaction between men and women based on religiosity is less than 0.05; the difference between the average of marital satisfaction is significant in different levels of adherence to religion by using analysis of variance in both women (P = 0.000, F = 7.484) and male (P = 0.001, F = 15.026). That religiosity in women and their partners can significantly predicted marital satisfaction between men and women.

## Discussion and conclusion

Adherence to the religion of the factors that have a significant role in marital satisfaction and proven in numerous studies as well as with religious teachings is confirmed. This study indicated that there was a direct link among men and women in religious commitment and adhere to the religion of the couple’s marital satisfaction was higher. The domestic research results as Thanagooii 2013 [**18**,**22**,**23**], Aghapour 2011 [**24**], and [**25**] has been consistent. In this regard, Khodayarifard et al (2007) showed a positive and notable link among marital satisfaction and attitudes. The marital satisfaction among those via religious restrictions very high, the highest among those with low religious restrictions, its lowest level. The findings indicated that the efficiency of cognitive behavioral team therapy with religious advice, compared to classical cognitive behavioral team therapy, enhancing the quality of the marital relationship of women, significantly contributed [**26**]. Sanagooii 2013 showed that religious women are less committed to the faultless models and ultimately have higher marital satisfaction. 

As well as numerous research abroad in relation to the role of religion and religious beliefs and religious commitment has been made with marital satisfaction. Including [**27**] in their study to evaluate the link among religious ideology, rituals and married person’s marital satisfaction in 228 and they came to the conclusion that there is a powerful link among marital satisfaction and religious practices that the research confirms.

Miner (2004) religious disagreements among family members, causing family problems and thus reduce the satisfaction and ultimately increase knows divorce [**28**]. The study showed that religious couples who are more verbal communication with each other in their marital relationships are more successful [**29**]. The role of religion and religious homogeneity paid the ultimate religion has an important role in marital satisfaction.

In Hanler’s research and Knchoz (2005) there is also a direct link among marital satisfaction and religiosity is achieved. Some researchers also showed that individuals with higher levels of religiosity are lower than those who are religious level, marital stability are more and more satisfied with their marriage (quoted from Davison and Jünger, 2012). Religion or belief is a very important factor in the married life quality and satisfaction is achieved. In this way, the beliefs and religious behaviors of two intrapersonal and interpersonal couples the affected villages.

In terms of the individual, enabling individual who respects the physiological, emotional, and cognitive control his anger, he helps in life conflicts accept responsibility for their actions and overall improvement is thinking. In terms of interpersonal, religious adherence provides conditions that the person considers God when he is angry.

In fact, according to the origin of life and the relationship between couples is the correct attitude change [**30**]. In explaining the findings can be said that religious commitment plays a significant role in improving relations between the partners and causes in the areas of parenting, recreation, performing bears, more common objectives pursued sex, and this agreement will ultimately lead to increased satisfaction [**31**]. According to the importance and influence of religious culture it is proposed to counselors and psychotherapists more Islamic approach because it is consistent with the culture of the country in order to increase the use of marital satisfaction, and skills training workshops for all couples before marriage is compulsory.

**Acknowledgement**

This paper is part of a MSc project in Midwifery and thereby we would like to thank all the professors from the Faculty of Nursing and Midwifery, Iran University of Medical Sciences, Tehran, and all the couples who took part in the research.
